# A Lyapunov Optimization-Based Approach to Autonomous Vehicle Local Path Planning

**DOI:** 10.3390/s24248031

**Published:** 2024-12-16

**Authors:** Ziba Arjmandzadeh, Mohammad Hossein Abbasi, Hanchen Wang, Jiangfeng Zhang, Bin Xu

**Affiliations:** 1 School of Aerospace and Mechanical Engineering, The University of Oklahoma, Norman, OK 73019, USA; ziba.arjmandzadeh-1@ou.edu (Z.A.); haw110@ou.edu (H.W.); 2 Department of Automotive Engineering, Clemson University, Greenville, SC 29607, USA; mabbasi@clemson.edu (M.H.A.); jiangfz@clemson.edu (J.Z.)

**Keywords:** autonomous vehicles, Lyapunov Optimization, path planning, model predictive control

## Abstract

Autonomous vehicles (AVs) offer significant potential to improve safety, reduce emissions, and increase comfort, drawing substantial attention from both research and industry. A critical challenge in achieving SAE Level 5 autonomy, full automation, is path planning. Ongoing efforts in academia and industry are focused on optimizing AV path planning, reducing computational complexity, and enhancing safety. This paper presents a novel approach using Lyapunov Optimization (LO) for local path planning in AVs. The proposed LO model is benchmarked against two conventional methods: model predictive control and a sampling-based approach. Additionally, an AV prototype was developed and tested in Norman, Oklahoma, where it collected data to evaluate the performance of the three control algorithms used in this study. To minimize costs and increase real-world applicability, a vision-only solution was employed for object detection and the generation of bird’s-eye-view coordinate data. Each control algorithm, i.e., Lyapunov Optimization (LO) and the two baseline methods, were independently used to generate safe and smooth paths for the AV based on the collected data. The approaches were then compared in terms of path smoothness, safety, and computation time. Notably, the proposed LO strategy demonstrated at least a 20 times reduction in computation time compared to the baseline methods.

## 1. Introduction

Developments in vehicle technology, along with advancements in driver assistant systems, have contributed to the advent of autonomous vehicles (AVs) [1]. AVs are gaining traction for their potential to enhance safety and reduce environmental pollution [2,3]. This paper focuses on AV local path planning. Local path planning, along with environment perception and control, construct the building blocks of AV intelligent behavior [4]. Furthermore, in this work, we generated a dataset utilizing an autonomous 2015 Nissan Leaf S in urban driving conditions. Conversely, quite a few existing studies exploit available datasets and concentrate on highway path planning [5,6,7].

AVs encounter various scenarios, including curved roads, intersections, obstacles, ramp merging, etc., that are explored in different papers such as [6,8,9,10]. These studies aim to find a safe, comfortable, and collision-free trajectory that pursues a reference line, considering the static and dynamic obstacles.

Various path planning methods are investigated in the literature [1], including sampling-based methods [11], model predictive control (MPC), and deep learning-based approaches. Sampling-based methods are widely used in tracking control problems, particularly in emulating human driving behavior. A key advantage of using sampling-based methods with Frenet coordinates is their ability to decouple lateral and longitudinal motions, which simplifies the mathematical representation of curved trajectories. Ref. [11] introduces a sampling-based method for optimal trajectory generation for the first time. Similarly, [5] utilizes a sampling-based approach within the Frenet frame for path planning while accounting for static obstacles. In [12], a sampling-based model is designed for collision-free path planning and validated through simulation. Further, sampling-based approaches using the Frenet frame generate multiple candidate trajectories at each time step and select the best one, which adds extra computation burden for practical applications.

MPC resolves redundant trajectory generation in sampling-based methods by directly finding the optimal path. In addition, MPC fortunately includes system dynamics and nonlinear and physical constraints in the underlying optimization problem. Moreover, the weighted cost function of MPC enables the customization of model performance and the reduction in energy consumption, facilitating the achievement of the optimal solution. MPC is employed in [13] for AV local path planning, where collision avoidance is obtained through potential fields and Farka’s lemma. The authors in [14] design a nonlinear MPC to solve the path-planning problem and shorten the computation time. Ref. [15] investigates a two-stage path-planning strategy where, while ignoring the obstacles, a local path is generated in the first stage. The second stage then optimizes ego vehicle maneuvers by MPC. Leveraging Frenet coordinates, a collision-free path is found by a gradient decent optimizer in [16]. Then, a PI-MPC controller follows the optimal path. The study in [17] proposes a joint Cartesian–Frenet MPC methodology, where the vehicle control model is outlined in the Cartesian space and road constraints are delineated in the Frenet coordinates. Then, the joint model is tested on a delivery and a passenger vehicle. An improved particle swarm optimization-based MPC method is developed in [18] for the cooperative control of local planning and path tracking for intelligent vehicles. Despite the advantages of MPC, acceptable control performance may require a prediction horizon that renders the slow implementation of MPC for practical applications.

Deep learning techniques address the high computational cost associated with sampling-based and optimization-based approaches. Unlike these methods, once a neural network is fully trained, deep learning enables rapid real-time implementation. Ref. [19] provides a comprehensive review of the latest advancements in deep learning-based path planning. Artificial neural networks are employed for various tasks such as collision avoidance, overtaking, safe navigation, and lane changing, as highlighted in studies [7,20,21,22]. However, several disadvantages emerge when applying deep learning to autonomous driving. One key issue is the lack of interpretability in neural networks, which can complicate legal accountability in the event of an accident. Additionally, deep learning algorithms require vast amounts of training data, limiting researchers to large datasets and creating challenges in obtaining or processing such data efficiently.

To overcome the challenges of high computational demands in sampling-based methods and model predictive control (MPC), as well as the large data requirements and lack of interpretability in deep learning approaches, this study proposes a novel solution based on the Lyapunov Optimization (LO) strategy. LO is recognized for its fast computational capabilities [23], and its mathematical foundation makes its behavior highly interpretable. This efficiency makes LO particularly suitable for accelerating AV path planning calculations. In this study, we leverage the LO approach to explore local path planning for AVs in dense urban traffic scenarios. To evaluate the performance of the proposed LO algorithm, we also introduce MPC and a sampling-based method as benchmark approaches for comparison.

Introduced in [24], the LO method is a relatively recent approach that decomposes an optimization problem into multiple subproblems, with each subproblem corresponding to a specific time step. The decomposition substantially increases the computation speed [23], while independence from other time steps relaxes the need for future information. Theorems 1 and 2 prove how the LO method closely follows the global optimal solution. Consequently, the computation complexity is diminished by downsizing the optimization problem. To the best of the authors’ knowledge, the LO method is not employed for AV local path planning, but it is used in close contexts that are reviewed in the following.

The LO method uses a Lyapunov function to optimally control a dynamical system. This Lyapunov function is positive definite and increases as the system nears instability. To maintain system stability, it is crucial to minimize the drift of the Lyapunov function. Furthermore, in [24], the original work that introduced Lyapunov Optimization (LO) uses Lyapunov drift for optimal control of queuing networks. A weighted penalty term is incorporated into the Lyapunov drift, forming the drift-plus-penalty framework. This approach ensures system stability while minimizing the penalty by minimizing the drift-plus-penalty expression. To apply the LO method to our problem, we transform it into a queue stability problem, as described in Section 2.3, by constructing virtual queues.

The authors in [25] employ the Lyapunov concept to investigate the problem of stability and synchronization of fractional-order complex-valued neural networks with time delay. In [26], the finite-time and fixed-time stability theorems of general fractional-order impulsive discontinuous systems through an indefinite Lyapunov functional approach are studied. In Ref. [27], the LO method is applied to traffic control, introducing a real-time global path planning technique aimed at optimizing the overall spatial utilization of the road network. The authors in [28] establish an online edge processing scheduling algorithm based on LO where unmanned aerial vehicle base stations are deployed as moving mobile edge computing servers. The proposed algorithm in [28] may face a path planning issue, which is tackled by reinforcement learning. Ref. [29] utilizes LO as a tool for exploring communication and localization of robotic networks in harsh environments, such as lunar, planetary, or subterranean environments. Lastly, the article in [30] also uses LO to deal with traffic control.

This paper introduces a novel local path planning approach based on the LO algorithm, chosen for its rapid computational efficiency. The LO method transforms the optimization problem into a queue stability problem by replacing system dynamics with virtual queues. Additionally, queue backlogs are incorporated into the objective function using a positive definite Lyapunov function, forming the drift-plus-penalty term. The LO minimizes the upper bound of the drift-plus-penalty term. This upper bound is first derived from the initial theorem and further refined by the second theorem, which proves that minimizing the upper bound results in a solution that approaches the global optimum. The contributions of our work are as follows:

- The problem of the local path planning of an AV is solved via LO for the first time;
- The optimization problem is transformed into a queue stability problem in order to apply LO;
- The standard LO algorithm is modified to change the desired state values dynamically from one time step to another;
- The proposed approach drastically reduces computation time.

The remaining sections of the paper are structured as follows. The problem formulation is extensively elaborated upon in Section 2. The experimental data collection is briefly explained in Section 3. In Section 4, numerical results are presented for three different scenes. Finally, the conclusions are drawn in Section 5.

## 2. Problem Formulation

This section defines the path planning problem and presents a solution approach using a sampling-based method illustrated in the Frenet frame, along with a description of the LO technique.

### 2.1. Environment

**Figure 1:**
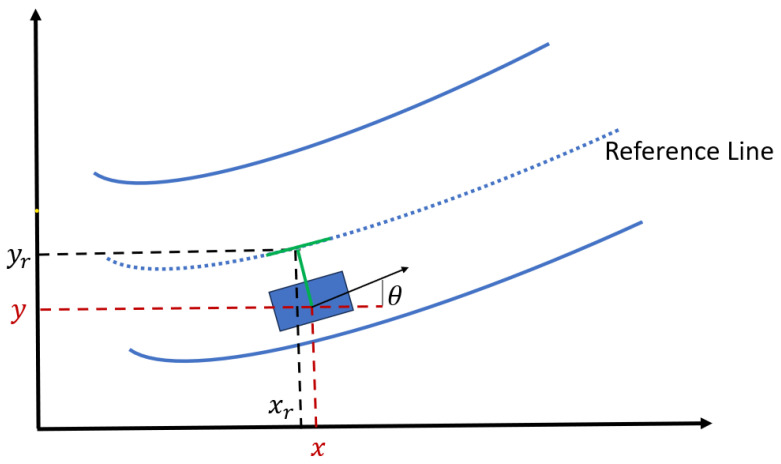
The geometry of the path.

Figure 1 illustrates an example of a car (the blue rectangle) on the road with a reference line to follow, where each point is denoted by (x,y) in the Cartesian system. Further, $\left(x_{r},y_{r}\right)$ indicates a point on the reference line closest to the vehicle’s center of gravity (CG), while the heading angle and vehicle’s velocity are represented by θ and *v*, respectively. The Euler approximation method is chosen for state equation discretization due to its widespread use and simplicity. However, it can result in coarse approximations when the sampling time is too large. To mitigate this limitation, this work reduces the sampling time to a level that strikes a balance between improved accuracy and acceptable computational cost for autonomous driving applications. The dynamic equation of the vehicle can be formulated as the following [31]:

(1) $$z_{k+1}=z_{k}+f\left(z_{k},c_{k}\right)\Delta t,$$

where subscript *k* is time step, Δt shows the sampling time, $c_{k}$ denotes input, and the vehicle’s state at *k* is $z_{k}={\left[\begin{array}{cccc} x_{k} & y_{k} & \theta_{k} & v_{k} \end{array}\right]}^{T}$. Moreover,

$$f\left(z_{k},c_{k}\right)={\left[\begin{array}{cccc} v_{k}\cos\theta_{k} & v_{k}\sin\theta_{k} & \omega_{k} & a_{k} \end{array}\right]}^{T},$$

where $\omega_{k}$ is angular velocity at *k*, $a_{k}$ indicates vehicle longitudinal acceleration at *k*, and $c_{k}={\left[\begin{array}{cc} \omega_{k} & a_{k} \end{array}\right]}^{T}$. Furthermore, environmental information, such as roadway waypoints, lane markings, and the positions of surrounding vehicles, is gathered through image processing using a real AV (see Section 3).

### 2.2. Path Planning Optimization Problem

**Figure 2:**
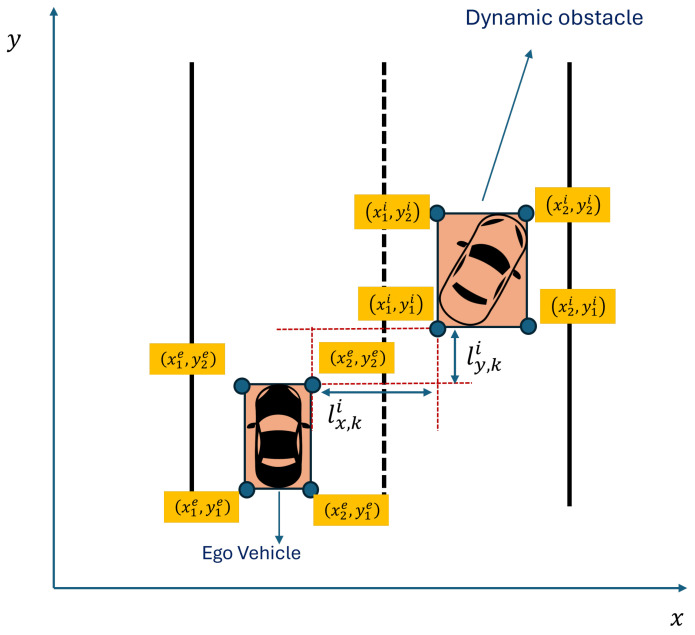
$r_{k}$ components $l_{x,k}^{i}$ and $l_{y,k}^{i}$.

The following nonlinear, constrained optimization problem is formulated to address the local path planning challenge, prioritizing safety and comfort for passengers while maintaining the desired speed.

(2a) $$\begin{array}{cc} \min\;\; & \frac{1}{N}E\left\{\sum_{k=1}^{N}(w_{gd}D_{k}^{2}+w_{ym}r_{k}+w_{v}{\left(v_{k}^{d}-v_{k}\right)}^{2}+\right.\\ \; & w_{a}a_{k}^{2}+w_{y}\omega_{k}^{2}+w_{j}{\left(a_{k}-a_{k-1}\right)}^{2})\} \end{array}$$

subject to

(2b) $$\begin{array}{cc} \; & \left[\begin{array}{c} x_{k+1}\\ y_{k+1}\\ \theta_{k+1}\\ v_{k+1} \end{array}\right]=\left[\begin{array}{c} x_{k}\\ y_{k}\\ \theta_{k}\\ v_{k} \end{array}\right]+\left[\begin{array}{c} v_{k}\cos\theta_{k}\\ v_{k}\sin\theta_{k}\\ \omega_{k}\\ a_{k} \end{array}\right]\Delta t,\;\forall k, \end{array}$$

(2c) $$\begin{array}{cc} \; & a^{\min}\leq a_{k}\leq a^{\max},\;\forall k, \end{array}$$

(2d) $$\begin{array}{cc} \; & \omega^{\min}\leq \omega_{k}\leq \omega^{\max},\;\forall k, \end{array}$$

where *N* is the total number of time steps; E{·} denotes expectation; $w_{gd}$, $w_{ym}$, $w_{v}$, $w_{a}$, $w_{y}$, and $w_{j}$ represent weights; $D_{k}$ expresses the Euclidian distance between the ego vehicle’s CG, $\left(x_{k},y_{k}\right)$ and $\left(x_{r},y_{r}\right)$ at time *k*. This term aims to minimize the reference position tracking error. $v_{k}^{d}$ presents the desired speed at *k*. This term aims to minimize the speed tracking error. $a^{\min}$ and $a^{\max}$ are acceleration boundaries. $\omega^{\min}$ and $\omega^{\max}$ show angular velocity boundaries. These acceleration and angular velocity terms aim to minimize the control input. ${\left(a_{k}-a_{k-1}\right)}^{2}$ is used to minimize longitudinal jerk. The barrier function $r_{k}$ is fashioned for collision avoidance as follows:

(3) $$\begin{array}{cc} r_{k} & =\sum_{i=1}^{N_{o}}\frac{1}{\eta_{x}l_{x,k}^{i}+\eta_{y}l_{y,k}^{i}}, \end{array}$$

(4) $$\begin{array}{cc} l_{x,k}^{i} & =\left\{\begin{array}{cc} x_{1}^{i}-x_{2}^{e}, & \mathrm{if}x_{2}^{e}<x_{1}^{i},\\ x_{1}^{e}-x_{2}^{i}, & \mathrm{if}x_{1}^{e}>x_{2}^{i},\\ 0, & O.W., \end{array}\right., \end{array}$$

(5) $$\begin{array}{cc} l_{y,k}^{i} & =\left\{\begin{array}{cc} y_{1}^{i}-y_{2}^{e}, & \mathrm{if}y_{2}^{e}<y_{1}^{i},\\ \infty , & \mathrm{if}y_{1}^{e}>y_{2}^{i},\\ 0, & O.W., \end{array}\right. \end{array}$$

where *i* is the obstacle index; *e* represents the ego vehicle; $N_{o}$ shows the total number of surrounding obstacles captured by image processing; $\eta_{x}$ and $\eta_{y}$ are weights; $l_{x,k}^{i}$ is the lateral distance of the ego vehicle from the obstacle at time *k* as displayed in Figure 2; $l_{y,k}^{i}$ indicates the longitudinal distance from the object; $x_{1}^{i}$, $x_{2}^{i}$, $y_{1}^{i}$, and $y_{2}^{i}$ are used to define the corners of the *i*-th obstacle’s bounding box; $x_{1}^{e}$, $x_{2}^{e}$, $y_{1}^{e}$, and $y_{2}^{e}$ locate the bounding box corners of the ego vehicle. The function $r_{k}$ is defined such that if a collision occurs with object *i*, $l_{x,k}^{i}=l_{y,k}^{i}=0$ and

$$\forall i:\;\lim_{\begin{array}{c} l_{x,k}^{i}\to 0\\ l_{y,k}^{i}\to 0 \end{array}}r_{k}=\infty .$$

### 2.3. Reformulation

For each of the states in (2b), a virtual queue is constructed as the following:

(6a) $$\begin{array}{cc} Q_{k}^{x} & =x_{k}-x_{k}^{d}, \end{array}$$

(6b) $$\begin{array}{cc} Q_{k}^{y} & =y_{k}-y_{k}^{d}, \end{array}$$

(6c) $$\begin{array}{cc} Q_{k}^{\theta } & =\theta_{k}-\theta_{k}^{d}, \end{array}$$

(6d) $$\begin{array}{cc} Q_{k}^{v} & =v_{k}-v_{k}^{d}, \end{array}$$

where $x_{k}^{d}$, $y_{k}^{d}$, $\theta_{k}^{d}$, and $v_{k}^{d}$ are desired state values. In detail, $\left(x_{k}^{d},y_{k}^{d}\right)$ are coordinates of the nearest waypoint (obtained from the reference line), $\theta_{k}^{d}$ is the desired steering angle based on the relative position of the vehicle and the reference line, and $v_{k}^{d}$ is the desired velocity. The queue length vector is defined as $Q_{k}=\left[Q_{k}^{x},Q_{k}^{y},Q_{k}^{\theta },Q_{k}^{v}\right]$. By combining (6) in (2b), we obtain the following:

(7a) $$\begin{array}{cc} Q_{k+1}^{x} & =Q_{k}^{x}+v_{k}\cos\theta_{k}\Delta t-x_{k+1}^{d}+x_{k}^{d}=Q_{k}^{x}+\xi_{k}^{x}, \end{array}$$

(7b) $$\begin{array}{cc} Q_{k+1}^{y} & =Q_{k}^{y}+v_{k}\sin\theta_{k}\Delta t-y_{k+1}^{d}+y_{k}^{d}=Q_{k}^{y}+\xi_{k}^{y}, \end{array}$$

(7c) $$\begin{array}{cc} Q_{k+1}^{\theta } & =Q_{k}^{\theta }+\omega_{k}\Delta t-\theta_{k+1}^{d}+\theta_{k}^{d}=Q_{k}^{\theta }+\xi_{k}^{\theta }, \end{array}$$

(7d) $$\begin{array}{cc} Q_{k+1}^{v} & =Q_{k}^{v}+a_{k}\Delta t-v_{k+1}^{d}+v_{k}^{d}=Q_{k}^{v}+\xi_{k}^{v}, \end{array}$$

where $\xi_{k}^{x}=v_{k}\cos\theta_{k}\Delta t-x_{k+1}^{d}+x_{k}^{d}$, $\xi_{k}^{y}=v_{k}\sin\theta_{k}\Delta t-y_{k+1}^{d}+y_{k}^{d}$, $\xi_{k}^{\theta }=\omega_{k}\Delta t-\theta_{k+1}^{d}+\theta_{k}^{d}$, and $\xi_{k}^{v}=a_{k}\Delta t-v_{k+1}^{d}+v_{k}^{d}$.

Using the virtual queues in (6), problem (2) can be transformed into a queue stability problem (8), where (2b) is changed to the stability of the queues.

(8a) $$\begin{array}{cc} \min\;\; & \frac{1}{N}E\left\{\sum_{k=1}^{N}(w_{gd}D_{k}^{2}+w_{ym}r_{k}+w_{v}{\left(v_{k}^{d}-v_{k}\right)}^{2}+\right.\\ \; & \left.w_{a}a_{k}^{2}+w_{y}\omega_{k}^{2}+w_{j}{\left(a_{k}-a_{k-1}\right)}^{2})\right\} \end{array}$$

subject to

(8b) $$\begin{array}{cc} \; & (2c),(2d)\\ \; & \mathrm{stability}\;\mathrm{of}\;\mathrm{virtual}\;\mathrm{queues}\;Q_{k}. \end{array}$$

Subsequently, the LO is applied to (8), which establishes an adaptive control policy for local path planning. Problem (8) is decomposed into *N* subproblems that are solved independently, while system stability is guaranteed.

### 2.4. Lyapunov Optimization Model

Define

(9) $$\begin{array}{ccc} u_{k} & =w_{gd}D_{k}^{2}+w_{ym}r_{k}+w_{v}{\left(v_{k}^{d}-v_{k}\right)}^{2} & \\ \; & +w_{a}a_{k}^{2}+w_{y}\omega_{k}^{2}+w_{j}{\left(a_{k}-a_{k-1}\right)}^{2}. \end{array}$$

Next, the Lyapunov function is defined as follows:

(10) $$L\left(Q_{k}\right)≜\frac{\beta_{1}}{2}{\left(Q_{k}^{x}\right)}^{2}+\frac{\beta_{2}}{2}{\left(Q_{k}^{y}\right)}^{2}+\frac{\beta_{3}}{2}{\left(Q_{k}^{\theta }\right)}^{2}+\frac{\beta_{4}}{2}{\left(Q_{k}^{v}\right)}^{2},$$

where $\beta_{1}$, $\beta_{2}$, $\beta_{3}$, and $\beta_{4}$ are positive constants. The Lyapunov drift is

(11) $$\Delta \left(Q_{k}\right)≜E\left\{L\left(Q_{k+1}\right)-L\left(Q_{k}\right)|Q_{k}\right\}.$$

Finally, the drift-plus-penalty term will be as follows [32]:

(12) $$\Delta \left(Q_{k}\right)+VE\left\{u_{k}|Q_{k}\right\}.$$

**Theorem 1.**  *At any time slot k, the Lyapunov drift-plus-penalty term has the following upper bound:*

(13) $$\begin{array}{cc} \; & \Delta \left(Q_{k}\right)+VE\left\{u_{k}|Q_{k}\right\}\leq B+\beta_{1}Q_{k}^{x}E\left\{\varrho_{k}^{x}|Q_{k}\right\}\\ \; & +\beta_{2}Q_{k}^{y}E\left\{\varrho_{k}^{y}|Q_{k}\right\}+\beta_{3}Q_{k}^{\theta }E\left\{\varrho_{k}^{\theta }|Q_{k}\right\}+\beta_{4}Q_{k}^{v}E\left\{\varrho_{k}^{v}|Q_{k}\right\}\\ \; & +VE\{u_{k}|Q_{k}\}, \end{array}$$

*where B is a positive constant shown in* (A11). *In addition, $\varrho_{k}^{x}$, $\varrho_{k}^{y}$, $\varrho_{k}^{\theta }$, and $\varrho_{k}^{v}$ are defined in* (A7).

**Proof.**  See Appendix A.    □

In Theorem 2, we will demonstrate the minimization of the upper bound of (13), instead of its left-hand side, for each time step *k* leads to a suboptimal solution which is close to the global optimal objective value of (2a), denoted as $p^{*}$. The convergence of the suboptimal solution to $p^{*}$ is controlled by coefficient *V*.

Before diving into Theorem 2, an optimization problem is formulated to minimize the upper bound of (13) as follows:

(14) $$\begin{array}{cc} \min\; & Vu_{k}+\beta_{1}Q_{k}^{x}\varrho_{k}^{x}+\beta_{2}Q_{k}^{y}\varrho_{k}^{y}+\beta_{3}Q_{k}^{\theta }\varrho_{k}^{\theta }+\beta_{4}Q_{k}^{v}\varrho_{k}^{v},\\ \; & \mathrm{subject}\;\mathrm{to}\;\;(2c)–(2d), \end{array}$$

where $a_{k}$ and $\omega_{k}$ are decision variables. Note that $Q_{k}^{x}$, $Q_{k}^{v}$, $Q_{k}^{\theta }$, and $Q_{k}^{v}$ are constants whose values are updated after solving (14) for time step *k* and before running (14) for time step k+1. Moreover, when (14) is being solved for k+1, the values of states at time step *k* are known. Therefore, according to (A7), the values of $\varrho_{k}^{x}$ and $\varrho_{k}^{y}$ are known, making terms $\beta_{1}Q_{k}^{x}\varrho_{k}^{x}$ and $\beta_{2}Q_{k}^{y}\varrho_{k}^{y}$ constants. Hence, solving (14) in its current form will not lead to the minimization of $Q_{k}^{x}$ and $Q_{k}^{y}$. In other words, by minimizing (14), the reference line is not tracked by the ego vehicle. This issue arises from the fact that $x_{k}$ and $y_{k}$ implicitly depend on $a_{k}$ and $\omega_{k}$. To resolve the issue, we propose using $v_{k+1}$ and $\theta_{k+1}$ instead of $v_{k}$ and $\theta_{k}$ in $\varrho_{k}^{x}$ and $\varrho_{k}^{y}$ as follows. Assume the following:

(15a) $$\begin{array}{cc} Q_{k}^{x}v_{k}\cos\theta_{k}\Delta t & \approx Q_{k}^{x}v_{k+1}\cos\theta_{k+1}\Delta t, \end{array}$$

(15b) $$\begin{array}{cc} Q_{k}^{y}v_{k}\sin\theta_{k}\Delta t & \approx Q_{k}^{y}v_{k+1}\sin\theta_{k+1}\Delta t. \end{array}$$

Utilizing (2b), $v_{k+1}$ and $\theta_{k+1}$ are substituted as

(16a) $$\begin{array}{cc} Q_{k}^{x}v_{k+1}\cos\theta_{k+1}\Delta t & =Q_{k}^{x}\left(v_{k}+a_{k}\Delta t\right)\cos\left(\theta_{k}+\omega_{k}\Delta t\right)\Delta t, \end{array}$$

(16b) $$\begin{array}{cc} Q_{k}^{y}v_{k+1}\sin\theta_{k+1}\Delta t & =Q_{k}^{y}\left(v_{k}+a_{k}\Delta t\right)\sin\left(\theta_{k}+\omega_{k}\Delta t\right)\Delta t. \end{array}$$

Finally, leveraging (A7) and plugging (16) in the objective function of (14), we obtain

(17) $$\begin{array}{cc} \; & \min\;Vu_{k}+\Lambda_{k},\\ & \mathrm{subject}\;\mathrm{to}\;(2c)–(2d) \end{array}$$

where

(18) $$\begin{array}{cc} \Lambda_{k} & =\beta_{1}Q_{k}^{x}\left(v_{k}+a_{k}\Delta t\right)\cos\left(\theta_{k}+\omega_{k}\Delta t\right)\Delta t\\ \; & +\beta_{2}Q_{k}^{y}\left(v_{k}+a_{k}\Delta t\right)\sin\left(\theta_{k}+\omega_{k}\Delta t\right)\Delta t+\beta_{3}Q_{k}^{\theta }\omega_{k}\Delta t\\ \; & +\beta_{4}Q_{k}^{v}a_{k}\Delta t. \end{array}$$

**Figure 3:**
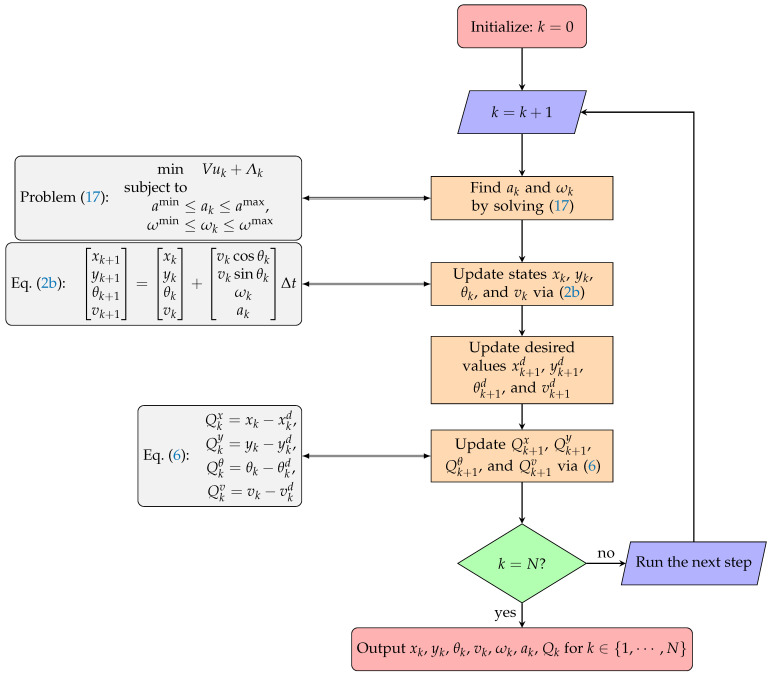
Algorithm 1 flowchart.

Problem (17) minimizes the upper bound of (13) for time step *k*. After each time step *k*, the queues should be updated before running problem (17) for the next time step k+1, as presented in Algorithm 1. Figure 3 shows the flowchart of Algorithm 1.
**Algorithm 1:** Lyapunov Optimization
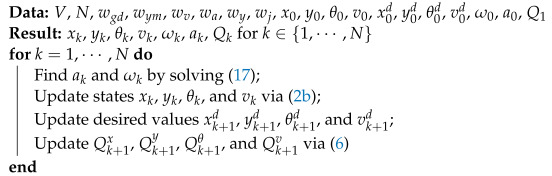

**Theorem 2.**  *For all N>1, the optimal solution of* (17), *denoted by $\left\{{\hat{a}}_{k},{\hat{\omega }}_{k}\right\}$, and the optimal solution of* (2), denoted by $\left\{a_{k}^{*},\omega_{k}^{*}\right\}$, satisfy the following inequality:

(19) $$\frac{1}{N}\sum_{k=1}^{N}E\left\{{\hat{u}}_{k}\right\}\leq \frac{B_{1}}{V}+p^{*}+\frac{1}{NV}E\left\{L({\hat{Q}}_{1})\right\},$$

*where $B_{1}$ is a constant defined in* (A30), $p^{*}$ *is the optimal objective value of* (2a), *and ${\hat{u}}_{k}$ is obtained by* (9) *using $\left\{{\hat{a}}_{k},{\hat{\omega }}_{k}\right\}$.*

**Proof.**  See Appendix B. □

Next, the problem is formulated for two baseline methods. Note that nonlinear problem (2) is directly tackled with a nonlinear MPC. Hence, the problem is only required to be reformulated for the sampling-based approach.

### 2.5. Sampling-Based Method

This method employs the Frenet frame, which utilizes Frenet coordinates to describe the position of any arbitrary point along a reference path through longitudinal and lateral displacements, denoted as *s* and *d*, respectively. It is important to note that the coordinates *s* and *d* are always perpendicular to each other at every point along the reference line. The approach consists of two main steps: (i) generating multiple candidate trajectories at each step and (ii) selecting the optimal path for the ego vehicle based on a cost function.

Using Frenet coordinates, we can decouple the lateral and longitudinal motion of the vehicle while simultaneously discretizing the driving state space to generate a set of target states. Based on the initial and target states, we create a series of trajectories for lateral and longitudinal motion, utilizing quintic and quartic polynomials, respectively. Additionally, a comprehensive cost function is formulated to evaluate comfort, safety, and deviation from the reference line. Furthermore, the variables *s* and *d* are represented by the following polynomials:

(20) $$\begin{array}{cc} d(t) & =c_{d_{0}}+c_{d_{1}}t+c_{d_{2}}t^{2}+c_{d_{3}}t^{3}+c_{d_{4}}t^{4}+c_{d_{5}}t^{5}, \end{array}$$

(21) $$\begin{array}{cc} s(t) & =c_{s_{0}}+c_{s_{1}}t+c_{s_{2}}t^{2}+c_{s_{3}}t^{3}+c_{s_{4}}t^{4}, \end{array}$$

where *t* is time and $c_{d_{x}}$ and $c_{s_{x}}$ are coefficients that should be calculated. Each trajectory starts at $t_{s}$ and ends at $t_{e}$ under the assumption that $t_{s}$ and $t_{e}$ are known. Considering Equations (20) and (21) and their time derivatives at the start and end points, the following set of equations is achieved:

(22) $$\begin{array}{cc} \left[\begin{array}{c} d(t_{s})\\ \dot{d}\left(t_{s}\right)\\ \ddot{d}\left(t_{s}\right)\\ d(t_{e})\\ \dot{d}\left(t_{e}\right)\\ \ddot{d}\left(t_{e}\right) \end{array}\right] & =\left[\begin{array}{cccccc} 1 & t_{s} & t_{s}^{2} & t_{s}^{3} & t_{s}^{4} & t_{s}^{5}\\ 0 & 1 & 2t_{s} & 3t_{s}^{2} & 4t_{s}^{3} & 5t_{s}^{4}\\ 0 & 0 & 2 & 6t_{s} & 12t_{s}^{2} & 20t_{s}^{3}\\ 1 & t_{e} & t_{e}^{2} & t_{e}^{3} & t_{e}^{4} & t_{e}^{5}\\ 0 & 1 & 2t_{e} & 3t_{e}^{2} & 4t_{e}^{3} & 5t_{e}^{4}\\ 0 & 0 & 2 & 6t_{e} & 12t_{e}^{2} & 20t_{e}^{3} \end{array}\right]\left[\begin{array}{c} c_{d_{0}}\\ c_{d_{1}}\\ c_{d_{2}}\\ c_{d_{3}}\\ c_{d_{4}}\\ c_{d_{5}} \end{array}\right], \end{array}$$

(23) $$\begin{array}{cc} \left[\begin{array}{c} s(t_{s})\\ \dot{s}\left(t_{s}\right)\\ \ddot{s}\left(t_{s}\right)\\ \dot{s}\left(t_{e}\right)\\ \ddot{s}\left(t_{e}\right) \end{array}\right] & =\left[\begin{array}{cccccc} 1 & t_{s} & t_{s}^{2} & t_{s}^{3} & t_{s}^{4} & t_{s}^{5}\\ 0 & 1 & 2t_{s} & 3t_{s}^{2} & 4t_{s}^{3} & 5t_{s}^{4}\\ 0 & 0 & 2 & 6t_{s} & 12t_{s}^{2} & 20t_{s}^{3}\\ 0 & 1 & 2t_{e} & 3t_{e}^{2} & 4t_{e}^{3} & 5t_{e}^{4}\\ 0 & 0 & 2 & 6t_{e} & 12t_{e}^{2} & 20t_{e}^{3} \end{array}\right]\left[\begin{array}{c} c_{s_{0}}\\ c_{s_{1}}\\ c_{s_{2}}\\ c_{s_{3}}\\ c_{s_{4}} \end{array}\right], \end{array}$$

which are used to find coefficients $c_{d_{x}}$ and $c_{s_{x}}$. To solve (22) and (23), assume $t_{s}=0$ and $t_{e}=\tau$, where τ is a positive constant. Therefore, by plugging $t_{s}=0$ into (22), we obtain $c_{d_{0}}=d\left(0\right)$, $c_{d_{1}}=\dot{d}\left(0\right)$, and $c_{d_{2}}=\ddot{d}\left(0\right)$. Consequently, the matrix Equation (22) simplifies into three equations and three unknowns. Note that we assume $\dot{d}\left(t_{e}\right)=\ddot{d}\left(t_{e}\right)=0$, while $d(t_{e})$ is selected such that the vehicle remains on the road. On the other hand, for (23), we have $c_{s_{0}}=s\left(0\right)$, $c_{s_{1}}=\dot{s}\left(0\right)$, and $c_{s_{2}}=\ddot{s}\left(0\right)$, which simplifies matrix Equation (23) to a two-by-two one. Moreover, $s(t_{s})$, $\dot{s}\left(t_{s}\right)$, and $\ddot{s}\left(t_{s}\right)$ are known from the previous step’s calculation, and the candidate trajectories are generated such that $\ddot{s}\left(t_{e}\right)=0$, and the $\dot{s}\left(t_{e}\right)$ value is selected close to the desired speed.

Accordingly, at each step, several trajectories are generated based on the combination of $d(t_{e})$ and $\dot{s}\left(t_{e}\right)$ values, and the best one is selected according to the cost function in (24). The candidate trajectories are generated by changing the values of

$$\begin{array}{cc} d(t_{e}) & \in \left[d\left(t_{s}\right)-LW,d\left(t_{s}\right)+LW\right],\\ \dot{s}\left(t_{e}\right) & \in \left[v_{k}^{d}-5/3.6,v_{k}^{d}+5/3.6\right], \end{array}$$

where LW is lane width. Finally, the cost function to be minimized is defined as

(24a) $$\begin{array}{cc} C & =C^{d}+C^{s}, \end{array}$$

(24b) $$\begin{array}{cc} C^{d} & =K_{j}\sum_{t=t_{s}}^{\tau }\dddot{d}{\left(t\right)}^{2}+K_{t}\tau +K_{d}d{\left(\tau \right)}^{2}, \end{array}$$

(24c) $$\begin{array}{cc} C^{s} & =K_{j}\sum_{t=t_{s}}^{\tau }\dddot{s}{\left(t\right)}^{2}+K_{t}\tau +K_{d}{\left(v_{k}^{d}-\dot{s}\left(t\right)\right)}^{2}, \end{array}$$

where $C^{d}$ and $C^{s}$ indicate lateral and longitudinal costs, and $K_{j}$, $K_{t}$, and $K_{d}$ are weights. The cost function aims to minimize both lateral and longitudinal jerks, reduce the lateral distance from the reference line, and ensure that the velocity matches the desired value.

## 3. Experimental Data Collection

**Figure 4:**
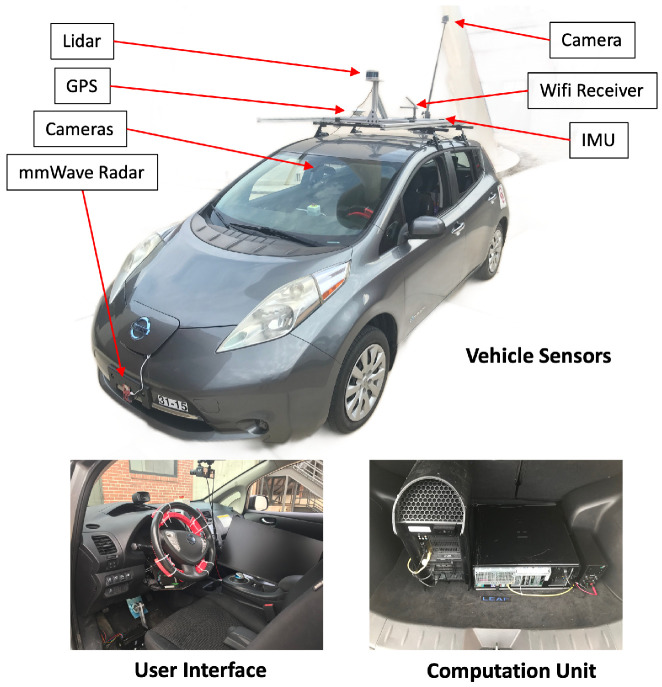
Environment perception sensors, human–machine interface, and computation unit of the Nissan Leaf test car at The University of Oklahoma Mobility and Intelligence Lab.

The data in this study were collected using a 2015 Nissan Leaf S battery electric vehicle in Norman, OK, USA. The test vehicle hardware configuration is shown in Figure 4. Environment perception sensors included Velodyne VLP-16 Puck lidar, Texas Instruments AWR1642BOOST mmWave radar, Swift Piski Multi GNSS GPS, Xsens MTi-30 IMU, and cameras. The steering wheel and two pedals were modified to achieve a drive-by-wire function. Two powerful computation stations were mounted on the trunk floor, including an Alienware R13 and a Dell T3500. Image data collected from the dashcam were utilized in this study. The camera data were broadcasted to the ROS2 Humble via a v4l2 camera driver and saved in an ROS bag. The image data were collected from Asp Ave for straight roads and 24th Ave NW for curly roads (Norman, OK, USA 73019). Three scenarios are analyzed in this study, with one straight road scenario (i.e., scenario 1) and two curly road scenarios (i.e., scenarios 2 and 3).

## 4. Simulation and Results

This section presents the results of local path planning using the proposed LO approach alongside two baseline methods for comparison. The key advantage of LO lies in its significantly faster computation speed. Rapid processing is crucial as the computation chip is one of the most costly pieces of hardware in AV setup. Moreover, the computations were executed on a computer with a 2.40 GHz Core i7 CPU with 20 cores and 16 GB RAM.

**Figure 5:**
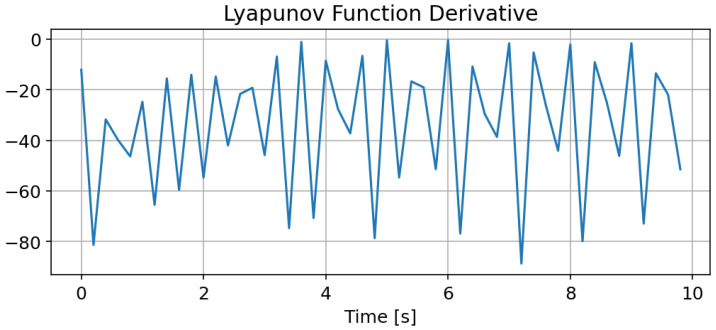
Scenario 1: Lyapunov function derivative.

First and foremost, the Lyapunov function derivative is plotted in Figure 5 to validate the proposed approach’s convergence. The convergence is verified as the derivative of the Lyapunov function in (10) and is negative.

**Figure 6:**
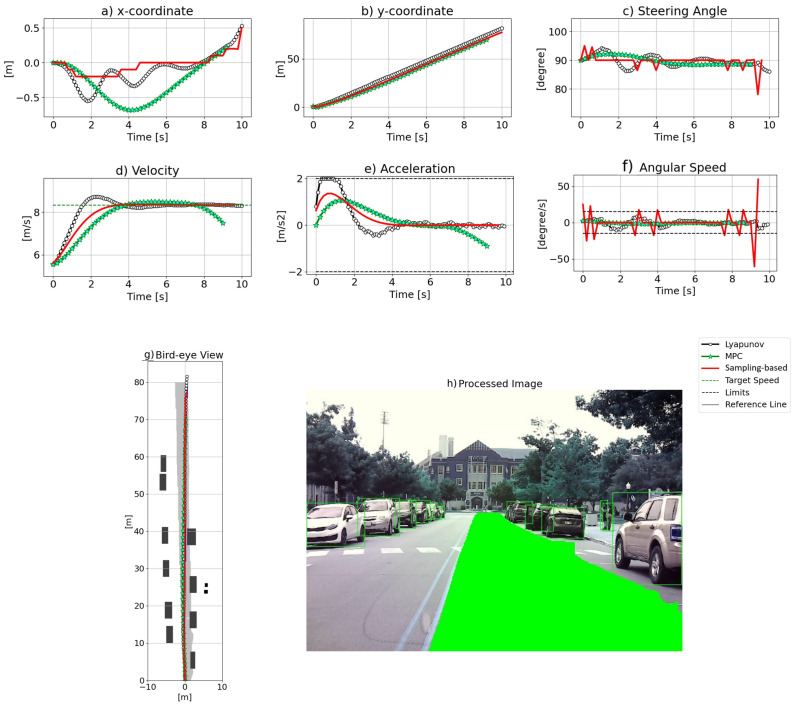
The results for scenario 1.

Figure 6 displays the results of scenario 1, which is straight road path planning in a narrow urban street where the lane is partially occupied by the vehicles parked next to the curb, as Figure 6h shows. The birds-eye-view image of the path ahead is generated and depicted in Figure 6g. According to Figure 6g, the paths generated by LO and the two baseline approaches are similar without significant disparity. MPC’s path is closest to the left-side solid lines, whereas the sampling-based method’s path is closest to the right side, where vehicles are parked, while the LO result falls in between the two baseline methods. Figure 6a illustrates the x-coordinates of the three algorithms. While MPC smoothly steers left to avoid parked cars and then right to follow the reference line, both LO and the sampling-based method exhibit less smooth transitions compared to MPC. Figure 6b demonstrates that the y-coordinates of all methods overlap and the three curves align well. On the other hand, Figure 6c,f represent steering angle and angular velocity results, respectively. Sharp changes in the steering angle observed in the sampling-based method are caused by significant variations in angular speed. These fluctuations arise because the sampling-based approach does not impose limits on the angular speed input. In contrast, the MPC and LO methods produce smooth results for both steering angle and velocity. Figure 6d,e show velocity and acceleration, respectively. LO exploits the maximum allowed acceleration to increase velocity to reach the desired value, whereas MPC and the sampling-based method show similar acceleration and velocity profiles. In the end, both the sampling-based algorithm and LO maintain the desired speed, while MPC experiences deceleration. This distinct behavior in MPC is attributed to its prediction horizon. As MPC anticipates the end of the road with no additional waypoints ahead, it proactively reduces speed.

**Figure 7:**
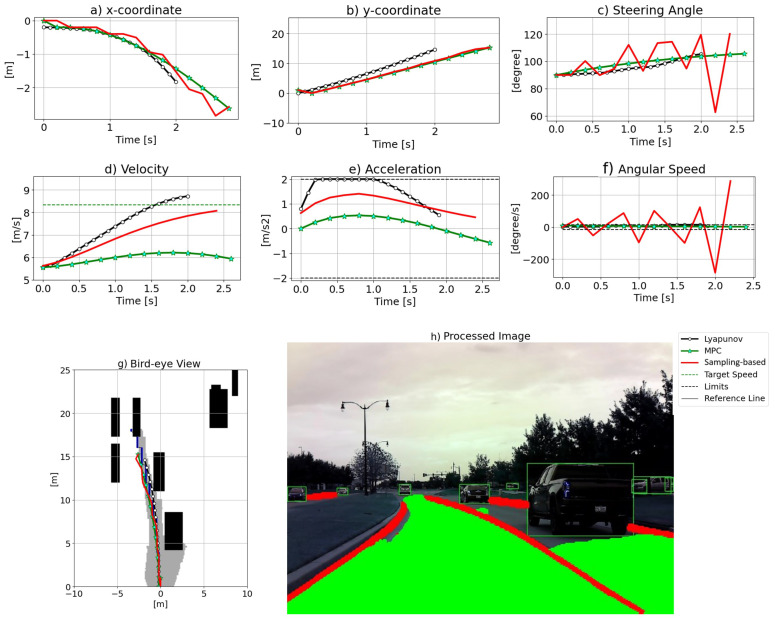
The results for scenario 2.

**Figure 8:**
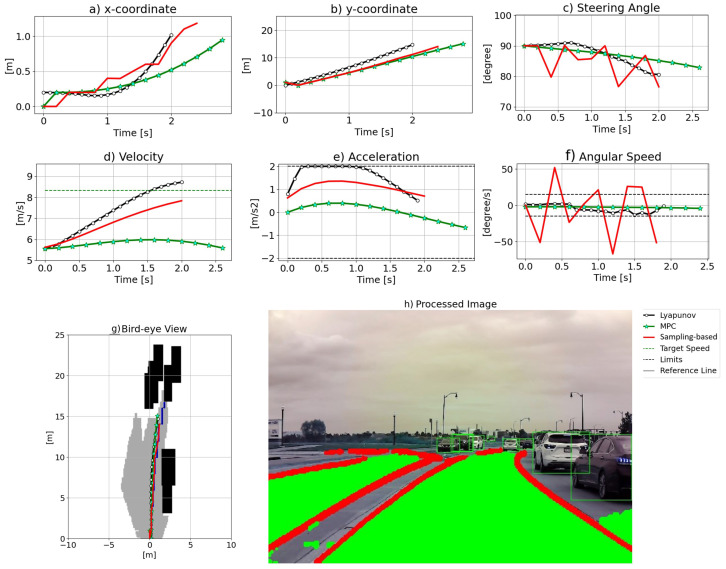
The results for scenario 3.

Figure 7 shows the second scenario on a three-lane street with a curve to the left. Based on Figure 7g, LO stays closer to the right edge of the lane, while MPC and the sampling-based approach maintain a larger lateral gap with the vehicle on the right (the sedan in front of the pickup truck in Figure 7h). Note that the step-wise solid black line in Figure 7g is the reference line. In Figure 7a,b, LO closely follows MPC and the sampling-based method results. Furthermore, Figure 7c,f demonstrate that LO and MPC exhibit much smoother handling of steering compared to the sampling-based method. As seen in Figure 7f, the sampling-based technique shows huge fluctuations. In addition, Figure 7d,e show that all approaches attempt to approach the desired speed. However, MPC decelerates shortly after the beginning of scenario 2 as a result of predicting the ego vehicle approaching the end of the path. Figure 8 presents the results for scenario 3, which is on a three-lane street with a curve to the right. As pictured in Figure 8g, the LO trajectory closely follows MPC’s, while the sampling-based method puts more effort into following the reference line, leading to non-smooth outcomes in subplots a, c, and f in Figure 8. Further, as shown in Figure 8d,e, MPC decelerates again to avoid passing the end point of the reference line.

The comparison of computation time, path smoothness, and safety among the three approaches is summarized in Table. Table 1. As shown in the table, to achieve a path generation task at a single time step, LO is at least faster than MPC by around 20 times and faster than the sampling-based method by 120 times. This computation time reduction by LO is significant. More importantly, it is achieved without safety and path smoothness compromise. The fast computation in LO, compared to MPC and the sampling-based approach, stems from the following. LO directly finds the optimal path, whereas the sampling-based method expends computational effort on analyzing multiple candidate trajectories. In comparison with MPC, LO only performs calculations for the current time step, while MPC runs the calculations for the next *c* steps, with *c* being the length of the control horizon. But only one step out of the *c* step calculations is executed, and then, MPC repeats everything for steps 2 to c+1, one of which will be executed next. In a sense, both MPC and the sampling-based methodology waste computation resources, generating paths that will not be realized. In contrast, all LO calculations are executed, making LO remarkably more efficient than MPC and the sampling-based method in terms of minimizing computation effort. Finally, in terms of path smoothness, LO and MPC attain a much smoother path compared to the sampling-based model. In addition, all approaches provide a safe trajectory as all paths are collision-free, and certain space margins are left against the obstacles.

## 5. Conclusions

This paper proposes a novel approach for AV local path planning using the LO algorithm. Besides perception and control, local path planning is an essential step in AV technology. The selection of the LO algorithm has its roots in the need for rapid computation in local path planning applications. After extensive mathematical derivations, the proposed algorithm boils down to Algorithm 1. In other words, Algorithm 1 is all one needs to implement the proposed strategy in this paper. The limitation of using LO is that Theorems 1 and 2 should be proved from scratch for each new problem. In order to compare the algorithms in the real-world problem, a 2015 Nissan Leaf S is made autonomous by installing the required sensors and controllers and is used to generate data by driving around urban streets in Norman, Oklahoma. The collected data are then fed to an image processing algorithm, which in turn provides the input data for Algorithm 1. Ultimately, the proposed approach is applied to three selected scenarios, and the results are compared with those of MPC and the sampling-based method. The results demonstrate that LO completes local path planning tasks with performance comparable to MPC, while being at least 20 times faster than MPC. Lastly, future studies can exploit this work by applying it to connected autonomous vehicle problems, where each AV is controlled by an LO algorithm while all LO algorithms interact with each other.

## Figures and Tables

**Table 1:** The computation time of one step (each step is Δt long), smoothness, and safety of the three approaches. The computation time is normalized. ‘Path Smootheness’ refers to the amplitude of angular velocity fluctuations.

Approaches	LO	MPC	Sampling-Based
Computation time	1	20	120
Path Smoothness	<20	<20	>50
Safety	No collision	No collision	No collision

## Data Availability

The raw data supporting the conclusions of this article will be made available by the authors on request.

## Appendix A. Proof of Theorem 1

**Proof.** Exploiting (10), we have

$$\begin{array}{cc} \; & L\left(Q_{k+1}\right)-L\left(Q_{k}\right)=\frac{\beta_{1}}{2}\left({\left(Q_{k+1}^{x}\right)}^{2}-{\left(Q_{k}^{x}\right)}^{2}\right)+\\ \; & \frac{\beta_{2}}{2}\left({\left(Q_{k+1}^{y}\right)}^{2}-{\left(Q_{k}^{y}\right)}^{2}\right)+\frac{\beta_{3}}{2}\left({\left(Q_{k+1}^{\theta }\right)}^{2}-{\left(Q_{k}^{\theta }\right)}^{2}\right)+\\ \; & \frac{\beta_{4}}{2}\left({\left(Q_{k+1}^{v}\right)}^{2}-{\left(Q_{k}^{v}\right)}^{2}\right). \end{array}$$

Using (6), we have

$$\begin{array}{cc} L\left(Q_{k+1}\right)-L\left(Q_{k}\right)= & \frac{\beta_{1}}{2}\left({\left(x_{k+1}-x_{k+1}^{d}\right)}^{2}-{\left(x_{k}-x_{k}^{d}\right)}^{2}\right)+\\ \; & \frac{\beta_{2}}{2}\left({\left(y_{k+1}-y_{k+1}^{d}\right)}^{2}-{\left(y_{k}-y_{k}^{d}\right)}^{2}\right)+\\ \; & \frac{\beta_{3}}{2}\left({\left(\theta_{k+1}-\theta_{k+1}^{d}\right)}^{2}-{\left(\theta_{k}-\theta_{k}^{d}\right)}^{2}\right)+\\ \; & \frac{\beta_{4}}{2}\left({\left(v_{k+1}-v_{k+1}^{d}\right)}^{2}-{\left(v_{k}-v_{k}^{d}\right)}^{2}\right), \end{array}$$

which can be expressed as

(A1) $$\begin{array}{cc} \; & L\left(Q_{k+1}\right)-L\left(Q_{k}\right)=\\ \; & \frac{\beta_{1}}{2}\left(x_{k+1}^{2}-x_{k}^{2}-2x_{k+1}^{d}x_{k+1}+2x_{k}^{d}x_{k}+{\left(x_{k+1}^{d}\right)}^{2}-{\left(x_{k}^{d}\right)}^{2}\right)+\\ \; & \frac{\beta_{2}}{2}\left(y_{k+1}^{2}-y_{k}^{2}-2y_{k+1}^{d}y_{k+1}+2y_{k}^{d}y_{k}+{\left(y_{k+1}^{d}\right)}^{2}-{\left(y_{k}^{d}\right)}^{2}\right)+\\ \; & \frac{\beta_{3}}{2}\left(\theta_{k+1}^{2}-\theta_{k}^{2}-2\theta_{k+1}^{d}\theta_{k+1}+2\theta_{k}^{d}\theta_{k}+{\left(\theta_{k+1}^{d}\right)}^{2}-{\left(\theta_{k}^{d}\right)}^{2}\right)+\\ \; & \frac{\beta_{4}}{2}\left(v_{k+1}^{2}-v_{k}^{2}-2v_{k+1}^{d}v_{k+1}+2v_{k}^{d}v_{k}+{\left(v_{k+1}^{d}\right)}^{2}-{\left(v_{k}^{d}\right)}^{2}\right). \end{array}$$

Define

(A2a) $$\begin{array}{cc} x_{k+1}^{d} & ≜x_{k}^{d}+\gamma_{k}^{x}, \end{array}$$

(A2b)
$$\begin{array}{cc} y_{k+1}^{d} & ≜y_{k}^{d}+\gamma_{k}^{y}, \end{array}$$

(A2c) $$\begin{array}{cc} \theta_{k+1}^{d} & ≜\theta_{k}^{d}+\gamma_{k}^{\theta }, \end{array}$$

(A2d) $$\begin{array}{cc} v_{k+1}^{d} & ≜v_{k}^{d}+\gamma_{k}^{v}. \end{array}$$

By plugging (A2) into (A1), we have

(A3) $$\begin{array}{cc} \; & L\left(Q_{k+1}\right)-L\left(Q_{k}\right)=\\ \; & \frac{\beta_{1}}{2}\left(x_{k+1}^{2}-x_{k}^{2}-2x_{k}^{d}\left(x_{k+1}-x_{k}\right)-2\gamma_{k}^{x}x_{k+1}+\Delta x_{k}^{d}\right)+\\ \; & \frac{\beta_{2}}{2}\left(y_{k+1}^{2}-y_{k}^{2}-2y_{k}^{d}\left(y_{k+1}-y_{k}\right)-2\gamma_{k}^{y}y_{k+1}+\Delta y_{k}^{d}\right)+\\ \; & \frac{\beta_{3}}{2}\left(\theta_{k+1}^{2}-\theta_{k}^{2}-2\theta_{k}^{d}\left(\theta_{k+1}-\theta_{k}\right)-2\gamma_{k}^{\theta }\theta_{k+1}+\Delta \theta_{k}^{d}\right)+\\ \; & \frac{\beta_{4}}{2}\left(v_{k+1}^{2}-v_{k}^{2}-2v_{k}^{d}\left(v_{k+1}-v_{k}\right)-2\gamma_{k}^{v}v_{k+1}+\Delta v_{k}^{d}\right), \end{array}$$

where $\Delta x_{k}^{d}={\left(x_{k+1}^{d}\right)}^{2}-{\left(x_{k}^{d}\right)}^{2}$, $\Delta y_{k}^{d}={\left(y_{k+1}^{d}\right)}^{2}-{\left(y_{k}^{d}\right)}^{2}$, $\Delta \theta_{k}^{d}={\left(\theta_{k+1}^{d}\right)}^{2}-{\left(\theta_{k}^{d}\right)}^{2}$, and $\Delta v_{k}^{d}={\left(v_{k+1}^{d}\right)}^{2}-{\left(v_{k}^{d}\right)}^{2}$. Note that at each time step *k*, a limited number of waypoints are detected by the mounted camera on the ego vehicle. Assume the farthest waypoint that can be detected by the camera is at $\left(X^{\max},Y^{\max}\right)$, where $X^{\max},Y^{\max}>0$. Therefore, $-X^{\max}\leq x_{k}^{d},\gamma_{k}^{x}\leq X^{\max}$ and $-Y^{\max}\leq y_{k}^{d},\gamma_{k}^{y}\leq Y^{\max}$ (The vehicle is always at (0,0).) Consequently, $\Delta x_{k}^{d}\leq {\left(X^{\max}\right)}^{2}$ and $\Delta y_{k}^{d}\leq {\left(Y^{\max}\right)}^{2}$. Moreover, $-2\gamma_{k}^{x}x_{k+1}\leq 2{\left(X^{\max}\right)}^{2}$ and $-2\gamma_{k}^{y}y_{k+1}\leq 2{\left(Y^{\max}\right)}^{2}$. On the other hand, $-\pi /2\leq \theta_{k}^{d},\gamma_{k}^{\theta }\leq \pi /2$. Hence, $\Delta \theta_{k}^{d}\leq \pi^{2}/4$ and $-2\gamma_{k}^{\theta }\theta_{k+1}\leq \pi^{2}/2$. Additionally, vehicle speed is limited to $V^{\max}$. Thus, $-V^{\max}\leq v_{k}^{d},\gamma_{k}^{v}\leq V^{\max}$, which yields $\Delta v_{k}^{d}\leq {\left(V^{\max}\right)}^{2}$ and $-2\gamma_{k}^{v}v_{k+1}\leq 2{\left(V^{\max}\right)}^{2}$. Finally, (A3) can be rewritten as

(A4) $$\begin{array}{cc} \; & L\left(Q_{k+1}\right)-L\left(Q_{k}\right)\leq \\ \; & \frac{\beta_{1}}{2}\left(x_{k+1}-x_{k}\right)\left(x_{k+1}+x_{k}-2x_{k}^{d}\right)+\zeta_{x}+\\ \; & \frac{\beta_{2}}{2}\left(y_{k+1}-y_{k}\right)\left(y_{k+1}+y_{k}-2y_{k}^{d}\right)+\zeta_{y}+\\ \; & \frac{\beta_{3}}{2}\left(\theta_{k+1}-\theta_{k}\right)\left(\theta_{k+1}+\theta_{k}-2\theta_{k}^{d}\right)+\zeta_{\theta }+\\ \; & \frac{\beta_{4}}{2}\left(v_{k+1}-v_{k}\right)\left(v_{k+1}+v_{k}-2v_{k}^{d}\right)+\zeta_{v}, \end{array}$$

where

$$\begin{array}{cc} \zeta_{x} & =\frac{3\beta_{1}}{2}{\left(X^{\max}\right)}^{2},\\ \zeta_{y} & =\frac{3\beta_{2}}{2}{\left(Y^{\max}\right)}^{2},\\ \zeta_{\theta } & =\frac{\beta_{3}}{2}\left(\frac{\pi^{2}}{4}+\frac{\pi^{2}}{2}\right),\\ \zeta_{v} & =\frac{3\beta_{4}}{2}{\left(V^{\max}\right)}^{2}. \end{array}$$

Noting that from (6) and (7), we have

(A5a) $$\begin{array}{cc} x_{k+1} & =x_{k}+\xi_{k}^{x}+x_{k+1}^{d}-x_{k}^{d}=x_{k}+\xi_{k}^{x}+\gamma_{k}^{x}, \end{array}$$

(A5b) $$\begin{array}{cc} y_{k+1} & =y_{k}+\xi_{k}^{y}+y_{k+1}^{d}-y_{k}^{d}=y_{k}+\xi_{k}^{y}+\gamma_{k}^{y}, \end{array}$$

(A5c) $$\begin{array}{cc} \theta_{k+1} & =\theta_{k}+\xi_{k}^{\theta }+\theta_{k+1}^{d}-\theta_{k}^{d}=\theta_{k}+\xi_{k}^{\theta }+\gamma_{k}^{\theta }, \end{array}$$

(A5d) $$\begin{array}{cc} v_{k+1} & =v_{k}+\xi_{k}^{v}+v_{k+1}^{d}-v_{k}^{d}=v_{k}+\xi_{k}^{v}+\gamma_{k}^{v}. \end{array}$$

Exploiting (A5), (A4) becomes

(A6) $$\begin{array}{cc} \; & L\left(Q_{k+1}\right)-L\left(Q_{k}\right)\leq \\ \; & \frac{\beta_{1}}{2}\left(\xi_{k}^{x}+\gamma_{k}^{x}\right)\left(\xi_{k}^{x}+\gamma_{k}^{x}+2x_{k}-2x_{k}^{d}\right)+\zeta_{x}+\\ \; & \frac{\beta_{2}}{2}\left(\xi_{k}^{y}+\gamma_{k}^{y}\right)\left(\xi_{k}^{y}+\gamma_{k}^{y}+2y_{k}-2y_{k}^{d}\right)+\zeta_{y}+\\ \; & \frac{\beta_{3}}{2}\left(\xi_{k}^{\theta }+\gamma_{k}^{\theta }\right)\left(\xi_{k}^{\theta }+\gamma_{k}^{\theta }+2\theta_{k}-2\theta_{k}^{d}\right)+\zeta_{\theta }+\\ \; & \frac{\beta_{4}}{2}\left(\xi_{k}^{v}+\gamma_{k}^{v}\right)\left(\xi_{k}^{v}+\gamma_{k}^{v}+2v_{k}-2v_{k}^{d}\right)+\zeta_{v}. \end{array}$$

Define

(A7a) $$\begin{array}{cc} \varrho_{k}^{x} & ≜\xi_{k}^{x}+\gamma_{k}^{x}=v_{k}\cos\theta_{k}\Delta t, \end{array}$$

(A7b) $$\begin{array}{cc} \varrho_{k}^{y} & ≜\xi_{k}^{y}+\gamma_{k}^{y}=v_{k}\sin\theta_{k}\Delta t, \end{array}$$

(A7c) $$\begin{array}{cc} \varrho_{k}^{\theta } & ≜\xi_{k}^{\theta }+\gamma_{k}^{\theta }=\omega_{k}\Delta t, \end{array}$$

(A7d) $$\begin{array}{cc} \varrho_{k}^{v} & ≜\xi_{k}^{v}+\gamma_{k}^{v}=a_{k}\Delta t. \end{array}$$

Substituting (A7) into (A6) and using (6), (A6) becomes

(A8) $$\begin{array}{cc} \; & L\left(Q_{k+1}\right)-L\left(Q_{k}\right)\leq \frac{\beta_{1}}{2}\varrho_{k}^{x}\left(\varrho_{k}^{x}+2Q_{k}^{x}\right)+\frac{\beta_{2}}{2}\varrho_{k}^{y}\left(\varrho_{k}^{y}+2Q_{k}^{y}\right)\\ \; & +\frac{\beta_{3}}{2}\varrho_{k}^{\theta }\left(\varrho_{k}^{\theta }+2Q_{k}^{\theta }\right)+\frac{\beta_{4}}{2}\varrho_{k}^{v}\left(\varrho_{k}^{v}+2Q_{k}^{v}\right)+\zeta , \end{array}$$

where $\zeta =\zeta_{x}+\zeta_{y}+\zeta_{\theta }+\zeta_{v}$, Equation (A8) is equal to

(A9) $$\begin{array}{cc} \; & L\left(Q_{k+1}\right)-L\left(Q_{k}\right)\leq \\ \; & \beta_{1}\left(\frac{1}{2}{\left(\varrho_{k}^{x}\right)}^{2}+Q_{k}^{x}\varrho_{k}^{x}\right)+\beta_{2}\left(\frac{1}{2}{\left(\varrho_{k}^{y}\right)}^{2}+Q_{k}^{y}\varrho_{k}^{y}\right)+\\ \; & \beta_{3}\left(\frac{1}{2}{\left(\varrho_{k}^{\theta }\right)}^{2}+Q_{k}^{\theta }\varrho_{k}^{\theta }\right)+\beta_{4}\left(\frac{1}{2}{\left(\varrho_{k}^{v}\right)}^{2}+Q_{k}^{v}\varrho_{k}^{v}\right)+\zeta . \end{array}$$

Since the vehicle’s speed is limited to $V^{\max}$, leveraging (A7), we have ${\left(\varrho_{k}^{x}\right)}^{2}\leq {\left(V^{\max}\Delta t\right)}^{2}$ and ${\left(\varrho_{k}^{y}\right)}^{2}\leq {\left(V^{\max}\Delta t\right)}^{2}$, respectively. Moreover, according to (2d), we have ${\left(\varrho_{k}^{\theta }\right)}^{2}\leq {\left(W\Delta t\right)}^{2}$, $W=\max\{|\omega^{\max}|,|\omega^{\min}|\}$. Analogously, based on (2c), we have ${\left(\varrho_{k}^{v}\right)}^{2}\leq {\left(A\Delta t\right)}^{2}$, $A=\max\{|a^{\max}|,|a^{\min}|\}$. Accordingly,

(A10) $$\begin{array}{cc} \; & L\left(Q_{k+1}\right)-L\left(Q_{k}\right)\leq \\ \; & \;\;B+\beta_{1}Q_{k}^{x}\varrho_{k}^{x}+\beta_{2}Q_{k}^{y}\varrho_{k}^{y}+\beta_{3}Q_{k}^{\theta }\varrho_{k}^{\theta }+\beta_{4}Q_{k}^{v}\varrho_{k}^{v}, \end{array}$$

where

(A11) $$B=\frac{\beta_{1}+\beta_{2}}{2}{\left(V^{\max}\Delta t\right)}^{2}+\frac{\beta_{3}}{2}{\left(W\Delta t\right)}^{2}+\frac{\beta_{4}}{2}{\left(A\Delta t\right)}^{2}+\zeta .$$

By taking conditional expectations from both hand sides with respect to $Q_{k}$, we have

$$\begin{array}{cc} \; & E\left\{L\left(Q_{k+1}\right)-L\left(Q_{k}\right)|Q_{k}\right\}\leq B+\beta_{1}Q_{k}^{x}E\left\{\varrho_{k}^{x}|Q_{k}\right\}+\\ \; & \beta_{2}Q_{k}^{y}E\left\{\varrho_{k}^{y}|Q_{k}\right\}+\beta_{3}Q_{k}^{\theta }E\left\{\varrho_{k}^{\theta }|Q_{k}\right\}+\beta_{4}Q_{k}^{v}E\left\{\varrho_{k}^{v}|Q_{k}\right\}, \end{array}$$

Utilizing (11) and adding $VE\{u_{k}|Q_{k}\}$ to both hand sides, we obtain (13)

$$\begin{array}{cc} \; & \Delta \left(Q_{k}\right)+VE\left\{u_{k}|Q_{k}\right\}\leq B+\beta_{1}Q_{k}^{x}E\left\{\varrho_{k}^{x}|Q_{k}\right\}\\ \; & +\beta_{2}Q_{k}^{y}E\left\{\varrho_{k}^{y}|Q_{k}\right\}+\beta_{3}Q_{k}^{\theta }E\left\{\varrho_{k}^{\theta }|Q_{k}\right\}+\beta_{4}Q_{k}^{v}E\left\{\varrho_{k}^{v}|Q_{k}\right\}\\ \; & +VE\{u_{k}|Q_{k}\}. \end{array}$$

□

## Appendix B. Proof of Theorem 2

Theorem 2 comprises the following steps:

- A relaxed version of problem (2) is constructed in (A13).
- It is shown that the optimal objective of (Appendix B), denoted as $\tilde{p}$, is less than or equal to the optimal objective of (2), $p^{*}$, i.e., $\tilde{p}\leq p^{*}$.
- (A13) satisfies the constraints of problem (17).
- The optimal solution of (17) is denoted by $\left\{{\hat{a}}_{k},{\hat{\omega }}_{k}\right\}$. Evaluating the objective of (17) at $\left\{{\hat{a}}_{k},{\hat{\omega }}_{k}\right\}$ yields $V{\hat{u}}_{k}+{\hat{\Lambda }}_{k}$.
- The objective of (17) evaluated at any values other than $\left\{{\hat{a}}_{k},{\hat{\omega }}_{k}\right\}$ is greater than or equal to $V{\hat{u}}_{k}+{\hat{\Lambda }}_{k}$.
- Evaluating the objective of (17) by the solution of (A13) yields $V{\hat{u}}_{k}+{\hat{\Lambda }}_{k}\leq V{\tilde{u}}_{k}+{\tilde{\Lambda }}_{k}$.
- With the help of mathematical manipulations, using $\tilde{p}\leq p^{*}$ and $V{\hat{u}}_{k}+{\hat{\Lambda }}_{k}\leq V{\tilde{u}}_{k}+{\tilde{\Lambda }}_{k}$, we derive $\frac{1}{N}\sum_{k=1}^{N}E\left\{{\hat{u}}_{k}\right\}\leq p^{*}+\frac{\mathrm{some}\;\mathrm{constants}}{V}$.

**Proof.** In (2b), by taking expectations on $v_{k+1}=v_{k}+a_{k}\Delta t$ and summing over $\left\{1,2,\cdots ,N\right\}$, we have

(A12) $$E\left\{v_{N+1}\right\}-E\left\{v_{1}\right\}=\sum_{k=1}^{N}E\left\{a_{k}\Delta t\right\},$$

where the left-hand side is obtained through the law of telescoping sums. All feasible solutions $\left\{a_{k},\omega_{k}\right\}$ that satisfy constrains of problem (2) also satisfy (A12). Define a new optimization problem by substituting $v_{k+1}=v_{k}+a_{k}\Delta t$ with (A12) as follows:

(A13a) $$\min\;\;\frac{1}{N}\sum_{k=1}^{N}E\left\{u_{k}\right\},$$

subject to

(2c)–(2d) ,(A12)

(A13b) $$\left[\begin{array}{c} x_{k+1}\\ y_{k+1}\\ \theta_{k+1} \end{array}\right]=\left[\begin{array}{c} x_{k}\\ y_{k}\\ \theta_{k} \end{array}\right]+\left[\begin{array}{c} v_{k}\cos\theta_{k}\\ v_{k}\sin\theta_{k}\\ \omega_{k} \end{array}\right]\Delta t,\;\forall k.$$

Since the expectation of sums is the sum of expectations, (A13a) is the same as (2a). Furthermore, as all feasible solutions $\left\{a_{k},\omega_{k}\right\}$ that satisfy constraints of problem (2) also satisfy constraints of (A13), problem (A13) is a relaxed version of problem (2). Consequently, the optimal solution of (2) is a feasible solution of (A13), and the optimal objective of (A13) is less than or equal to the optimal objective of (2). Suppose $\tilde{p}$ represents the optimal objective value of (A13) obtained from optimal solution $\left\{\tilde{a_{k}},\tilde{\omega_{k}}\right\}$; therefore, $\tilde{p}\leq p^{*}$,

(A14) $$\tilde{p}=\frac{1}{N}\sum_{k=1}^{N}E\left\{{\tilde{u}}_{k}\right\}\leq p^{*}=\frac{1}{N}\sum_{k=1}^{N}E\left\{u_{k}^{*}\right\},$$

where ${\tilde{u}}_{k}$ and $u_{k}^{*}$ are obtained from the optimal solutions of (A13) and (2) using (9), respectively. Additionally, since (17) minimizes the upper bound of the drift-plus-penalty term, the solutions of (17) yield the minimum of $Vu_{k}+\Lambda_{k}$ as

(A15) $$V{\hat{u}}_{k}+{\hat{\Lambda }}_{k},$$

where ${\hat{u}}_{k}$ and ${\hat{\Lambda }}_{k}$ are evaluated at $\left\{{\hat{a}}_{k},{\hat{\omega }}_{k}\right\}$. Evaluating $Vu_{k}+\Lambda_{k}$ at any other values satisfying the constraints of (17) yields a value greater than or equal to (A15). The solutions of the relaxed problem (A13) satisfy constraints of (17); therefore,

(A16) $$V{\hat{u}}_{k}+{\hat{\Lambda }}_{k}\leq V{\tilde{u}}_{k}+{\tilde{\Lambda }}_{k},$$

where ${\tilde{u}}_{k}$ and ${\tilde{\Lambda }}_{k}$ are evaluated at $\left\{{\tilde{a}}_{k},{\tilde{\omega }}_{k}\right\}$. By taking expectations with respect to ${\hat{Q}}_{t}$ of (A15),

(A17) $$VE\left\{{\hat{u}}_{k}|{\hat{Q}}_{k}\right\}+E\left\{{\hat{\Lambda }}_{k}|{\hat{Q}}_{k}\right\},$$

where

(A18) $$\begin{array}{cc} E\left\{{\hat{\Lambda }}_{k}|{\hat{Q}}_{k}\right\} & =\beta_{1}{\hat{Q}}_{k}^{x}E\left\{\left({\hat{v}}_{k}+{\hat{a}}_{k}\Delta t\right)\cos\left({\hat{\theta }}_{k}+{\hat{\omega }}_{k}\Delta t\right)\Delta t|{\hat{Q}}_{k}\right\}\\ \; & +\beta_{2}{\hat{Q}}_{k}^{y}E\left\{\left({\hat{v}}_{k}+{\hat{a}}_{k}\Delta t\right)\sin\left({\hat{\theta }}_{k}+{\hat{\omega }}_{k}\Delta t\right)\Delta t|{\hat{Q}}_{k}\right\}\\ \; & +\beta_{3}{\hat{Q}}_{k}^{\theta }E\left\{{\hat{\omega }}_{k}\Delta t|{\hat{Q}}_{k}\right\}+\beta_{4}{\hat{Q}}_{k}^{v}E\left\{{\hat{a}}_{k}\Delta t|{\hat{Q}}_{k}\right\}. \end{array}$$

By substituting (A17) into (13), under assumption (15), we have

(A19) $$\begin{array}{cc} \; & \Delta \left({\hat{Q}}_{k}\right)+VE\left\{{\hat{u}}_{k}|{\hat{Q}}_{k}\right\}\leq B+\beta_{1}{\hat{Q}}_{k}^{x}E\left\{{\hat{\varrho }}_{k}^{x}|{\hat{Q}}_{k}\right\}\\ \; & +\beta_{2}{\hat{Q}}_{k}^{y}E\left\{{\hat{\varrho }}_{k}^{y}|{\hat{Q}}_{k}\right\}+\beta_{3}{\hat{Q}}_{k}^{\theta }E\left\{{\hat{\varrho }}_{k}^{\theta }|{\hat{Q}}_{k}\right\}+\beta_{4}{\hat{Q}}_{k}^{v}E\left\{{\hat{\varrho }}_{k}^{v}|{\hat{Q}}_{k}\right\}\\ \; & +VE\{{\hat{u}}_{k}|{\hat{Q}}_{k}\}. \end{array}$$

By substituting ${\hat{\varrho }}_{k}^{x}$, ${\hat{\varrho }}_{k}^{y}$, ${\hat{\varrho }}_{k}^{\theta }$, and ${\hat{\varrho }}_{k}^{v}$ from (A7) and using assumption (15), Equation (A19) can be equivalently expressed as

(A20) $$\Delta \left({\hat{Q}}_{k}\right)+VE\left\{{\hat{u}}_{k}|{\hat{Q}}_{k}\right\}\leq B+E\left\{{\hat{\Lambda }}_{k}|{\hat{Q}}_{k}\right\}+VE\left\{{\hat{u}}_{k}|{\hat{Q}}_{k}\right\}.$$

By taking expectations from both hand sides, we have

(A21) $$E\left\{\Delta ({\hat{Q}}_{k})\right\}+VE\left\{{\hat{u}}_{k}\right\}\leq B+E\left\{{\hat{\Lambda }}_{k}\right\}+VE\left\{{\hat{u}}_{k}\right\},$$

where the condition with respect to ${\hat{Q}}_{k}$ is removed according to the law of iterated expectations, and

(A22) $$\begin{array}{cc} E\left\{{\hat{\Lambda }}_{k}\right\} & =\beta_{1}E\left\{{\hat{Q}}_{k}^{x}\right\}E\left\{\left({\hat{v}}_{k}+{\hat{a}}_{k}\Delta t\right)\cos\left({\hat{\theta }}_{k}+{\hat{\omega }}_{k}\Delta t\right)\Delta t\right\}\\ \; & +\beta_{2}E\left\{{\hat{Q}}_{k}^{y}\right\}E\left\{\left({\hat{v}}_{k}+{\hat{a}}_{k}\Delta t\right)\sin\left({\hat{\theta }}_{k}+{\hat{\omega }}_{k}\Delta t\right)\Delta t\right\}\\ \; & +\beta_{3}E\left\{{\hat{Q}}_{k}^{\theta }\right\}E\left\{{\hat{\omega }}_{k}\Delta t\right\}+\beta_{4}E\left\{{\hat{Q}}_{k}^{v}\right\}E\left\{{\hat{a}}_{k}\Delta t\right\}. \end{array}$$

Utilizing (A16) and (A21), we have

(A23) $$\begin{array}{cc} E\left\{\Delta ({\hat{Q}}_{k})\right\}+VE\left\{{\hat{u}}_{k}\right\} & \leq B+E\left\{{\hat{\Lambda }}_{k}\right\}+VE\left\{{\hat{u}}_{k}\right\}\\ \; & \leq B+E\left\{{\tilde{\Lambda }}_{k}\right\}+VE\left\{{\tilde{u}}_{k}\right\}. \end{array}$$

Using (11) and summing over all time steps,

(A24) $$\begin{array}{cc} \; & E\left\{L({\hat{Q}}_{N+1})\right\}-E\left\{L({\hat{Q}}_{1})\right\}+V\sum_{k=1}^{N}E\left\{{\hat{u}}_{k}\right\}\leq \\ \; & \;\;\;NB+\sum_{k=1}^{N}E\left\{{\tilde{\Lambda }}_{k}\right\}+V\sum_{k=1}^{N}E\left\{{\tilde{u}}_{k}\right\}. \end{array}$$

As $L(Q_{k})$ is a positive definite function, $E\left\{L({\hat{Q}}_{N+1})\right\}$ can be omitted, as follows:

(A25) $$\begin{array}{cc} \; & -E\left\{L({\hat{Q}}_{1})\right\}+V\sum_{k=1}^{N}E\left\{{\hat{u}}_{k}\right\}\leq \\ \; & \;\;\;NB+\sum_{k=1}^{N}E\left\{{\tilde{\Lambda }}_{k}\right\}+V\sum_{k=1}^{N}E\left\{{\tilde{u}}_{k}\right\}. \end{array}$$

By dividing both hand sides by NV and rearranging the following:

(A26) $$\begin{array}{cc} \; & \frac{1}{N}\sum_{k=1}^{N}E\left\{{\hat{u}}_{k}\right\}\leq \frac{B}{V}+\\ \; & \;\;\;\frac{1}{NV}\sum_{k=1}^{N}E\left\{{\tilde{\Lambda }}_{k}\right\}+\frac{1}{N}\sum_{k=1}^{N}E\left\{{\tilde{u}}_{k}\right\}+\frac{1}{NV}E\left\{L({\hat{Q}}_{1})\right\}. \end{array}$$

Based on the limits defined in order to construct inequality (A4), the maximum value of $\Lambda_{k}$ in (18) is

(A27) $$\begin{array}{cc} \Lambda_{k} & \leq \Lambda^{\max},\\ \Lambda^{\max} & =\left(\beta_{1}X^{\max}+\beta_{2}Y^{\max}\right)\left(V^{\max}+A\Delta t\right)\Delta t\\ \; & +\beta_{3}\frac{\pi }{2}W\Delta t+\beta_{4}V^{\max}A\Delta t. \end{array}$$

Therefore,

(A28) $$\begin{array}{cc} \; & \frac{1}{N}\sum_{k=1}^{N}E\left\{{\hat{u}}_{k}\right\}\leq \frac{B}{V}+\\ \; & \;\;\;\frac{\Lambda^{\max}}{V}+\frac{1}{N}\sum_{k=1}^{N}E\left\{{\tilde{u}}_{k}\right\}+\frac{1}{NV}E\left\{L({\hat{Q}}_{1})\right\}. \end{array}$$

Using (A14), we arrive at

(A29) $$\begin{array}{cc} \frac{1}{N}\sum_{k=1}^{N}E\left\{{\hat{u}}_{k}\right\} & \leq \frac{B_{1}}{V}+p^{*}+\frac{1}{NV}E\left\{L({\hat{Q}}_{1})\right\}, \end{array}$$

(A30) $$\begin{array}{cc} B_{1} & =B+\Lambda^{\max}. \end{array}$$

If V→∞, the left-hand side of (A29) converges to $p^{*}$. □
